# Improving the accuracy of blood pressure measuring devices in Australia: a modelled return on investment study

**DOI:** 10.1038/s41371-023-00866-2

**Published:** 2023-11-08

**Authors:** Zachary Desson, James E. Sharman, Andrew Searles, Aletta E. Schutte, Christian Delles, Michael Hecht Olsen, Pedro Ordunez, Alexis Hure, Rachael Morton, Gemma Figtree, Jacqui Webster, Garry Jennings, Julie Redfern, Stephen J. Nicholls, Martin McNamara, Simon Deeming, Kerry Doyle, Shanthi Ramanathan

**Affiliations:** 1 Health Research Economics, Hunter Medical Research Institute, Newcastle, NSW Australia; 2 Menzies Institute for Medical Research, University of Tasmania, Hobart, TAS Australia; 3 College of Health, Medicine and Wellbeing, School of Medicine and Public Health, University of Newcastle, Newcastle, NSW Australia; 4 School of Population Health, University of New South Wales, Sydney, NSW Australia; 5 Institute of Cardiovascular and Medical Sciences, University of Glasgow, Glasgow, UK; 6 Department of Medicine, University of Southern Denmark, Odense, Denmark; 7 Department of Non-Communicable Diseases and Mental Health, Pan American Health Organization, Washington, DC USA; 8 Faculty of Medicine and Health, University of Sydney, Sydney, NSW Australia; 9 Australian Cardiovascular Alliance, Chittaway Bay, NSW Australia; 10 The George Institute for Global Health, Sydney, NSW Australia; 11 Faculty of Medicine, Nursing and Health Sciences, Monash University, Melbourne, VIC Australia; 12 Victorian Heart Institute, Monash University, Clayton, VIC Australia; 13 The Sax Institute, Sydney, NSW Australia

**Keywords:** Renovascular hypertension, Health care

## Abstract

The VALID BP project was initiated to increase the availability of validated blood pressure measuring devices (BPMDs). The goal is to eliminate non validated BPMDs and minimise over- and underdiagnosis of hypertension caused by inaccurate readings. This study was undertaken to assess the potential return on investment in the VALID BP project. The Framework to Assess the Impact of Translational Health Research was applied to the VALID BP project. This paper focuses on the implementation of the cost benefit analysis aspect of this framework to monetise past research investment and model future research costs, implementation costs, and benefits. Analysis was based on reasoned assumptions about potential impacts from availability and use of validated BPMDs (assuming an end goal of 100% validated BPMDs available in Australia by 2028) and improved skills leading to more accurate BP measurement. After 5 years, with 20% attribution of benefits, there is a potential $1.14–$1.30 return for every dollar spent if the proportion of validated BPMDs and staff trained in proper BP measurement technique increased from 20% to 60%. After eight years (2020–2028) and assuming universal validation and training coverage, the returns would be between $2.70 and $3.20 per dollar spent (not including cost of side effects of unnecessary medication or downstream patient impacts from unmanaged hypertension). This modelled economic analysis indicates there will be positive downstream economic benefits if the availability of validated BPMDs is increased. The findings support ongoing efforts toward a universal regulatory framework for BPMDs and can be considered within more detailed future economic analyses.

## Introduction

High blood pressure (BP), clinically known as hypertension, affects ~1 in 3 adults in Australia and is a major risk factor for cardiovascular disease [1]. Effective management, including medication and lifestyle changes, can lower avoidable risk and prevent disease progression. Accurate and timely detection and diagnosis is critical in mitigating the long-term health impacts of hypertension. Effective management, including medication and lifestyle changes, can lower avoidable risk and prevent severe disease progression. Conversely, inaccurate BP measurement can lead to missed or improper diagnoses and management resulting in unnecessary medication use or avoidable downstream healthcare costs and negative health outcomes [2]. Under Australian clinical practice guidelines and most international guidelines, the threshold for hypertension diagnosis is ≥140 and/or 90 mmHg assessed based on at least two measurements taken one week apart [3, 4]. Given the importance of these readings to an accurate diagnosis and management, including treatment goal, the BP measuring device (BPMD) being used must provide accurate and reliable results.

An important step towards ensuring the accuracy and reliability of BPMDs is a validation study, by which devices are thoroughly tested using a standardised and acceptable protocol [5, 6]. However, most BPMDs (up to 85%) on the market are not appropriately validated in Australia [7–9]. The importance of validation was confirmed by a study evaluating 870 automated BPMDs. Validated devices provided readings within 4 mmHg of manual auscultatory BP 68% of the time, compared to only 15% of the time for non-validated devices [10]. Thus using non-validated BPMDs can introduce greater measurement variability, potentially leading to a missed or improper diagnosis, especially where BP is close to diagnostic and treatment thresholds [11–13]. Given the lack of a regulatory framework for the exclusive use of validated BPMDs in Australia, this gap in accuracy is of particular concern, as only 14.5% of automated upper arm and wrist cuff BPMDs available for online purchase have undergone appropriate validation testing [14].

In 2019, the Lancet Commission on Hypertension Group outlined a series of recommended actions to increase the global availability of appropriately validated BPMDs [15]. The Lancet Group conceived the VALID BP Project to implement these actions and work towards universal validation of all BPMDs available for purchase and use [16]. In addition to publishing articles in high-impact peer-reviewed journals, the VALID BP project has undertaken translational activities to effect regulatory change, such as producing and disseminating policy briefs and media releases. The VALID BP team have also developed practical resources, such as an online certification course for BP measurement, and their educational resources on good BP measurement technique were promoted through national and international networks including organisations like the World Hypertension League [17]. These activities were complemented by engagement with industry stakeholders to establish their perspectives on the issue and their role in validating all devices [18]. Advocacy was also broadly applied to influence regulatory agencies to prioritise the supply of validated BPMDs, both in Australia and globally [19–21].

In 2020, the VALID BP project was selected as an exemplar to be featured in online training materials to build capability in research impact assessment and the application of the Framework to Assess the Impact from Translational health research (FAIT) [22]. FAIT is a comprehensive impact assessment framework designed to be used prospectively to encourage greater translation of research and reporting on research impact. FAIT uses three validated methods that collectively produce a comprehensive multidimensional assessment of the impact of research that resonates with a wide range of stakeholders. Alongside a quantitative assessment of impact using metrics grouped under key domains of benefit, and a narrative account of the project’s impact (both available in the supplementary materials), FAIT also includes an economic analysis to estimate the return on investment (ROI) from the associated research. This economic analysis enriches the assessment by addressing the questions usually asked by funders, policymakers, and implementers of research about: (1) the full economic cost of conducting the research, (2) the resource commitments to implement the findings into practice and (3) the expected benefits from these research investments.

The aims of the study, as reported in this paper, were to:

1. Undertake a cost-benefit analysis of the VALID BP project to model returns on investment (both current and future) to assess the project’s economic impact at varying levels of projected effectiveness.
2. Generate evidence to inform whether continued investment in the research is likely to generate economic benefits that are socially meaningful.
3. Create a replicable protocol for this economic analysis that could be employed in the future for a re-evaluation of the ROI from VALID BP.
4. Identify challenges and limitations in the analysis to inform others looking to undertake similar analyses in the future.

## Methods

### Cost-benefit analysis

Several economic evaluation techniques can contribute to calculating ROI but the one selected for this study was a cost-benefit analysis (CBA). A CBA is the basis of an encompassing ROI analysis and is suggested as an appropriate technique for understanding the returns on research investment using FAIT [22]. It takes a broader societal perspective of the range of benefits captured and reported, both costs and consequences are reported in monetary values, and the reportable metric of CBA is a ratio of benefit per dollar of cost [23]. The single ratio form of reporting ROI allows direct comparison with ROI calculated for other programmes and can also be calculated using simulations or projections; both reasons for the selection of a CBA over other techniques for this study. These projections can then be compared to subsequent recalculations using actual data as the VALID BP programme progresses and delivers outputs. All monetary values used refer to the Australian dollar unless otherwise specified.

#### Inclusions and exclusions

The CBA included:

- The costs of VALID BP’s initial research and ongoing translational activities
- The costs associated with validating BPMDs and training healthcare practitioners in proper BP measurement technique
- The benefits of improved diagnostic accuracy for hypertension across the Australian healthcare system including cost savings from elimination of unnecessary medication use and prevention of disease sequalae and averted losses to economic productivity through better BP measurement and management.

The CBA excluded:

- The costs of replacing nonvalidated BPMDs
- Consumer price index (CPI) increases (inflation) in future research and implementation costs
- The potential price differences between validated and non-validated devices
- The cost of treatment (mainly medications) for previously undiagnosed patients

### Program Logic Model

**Fig. 1:**
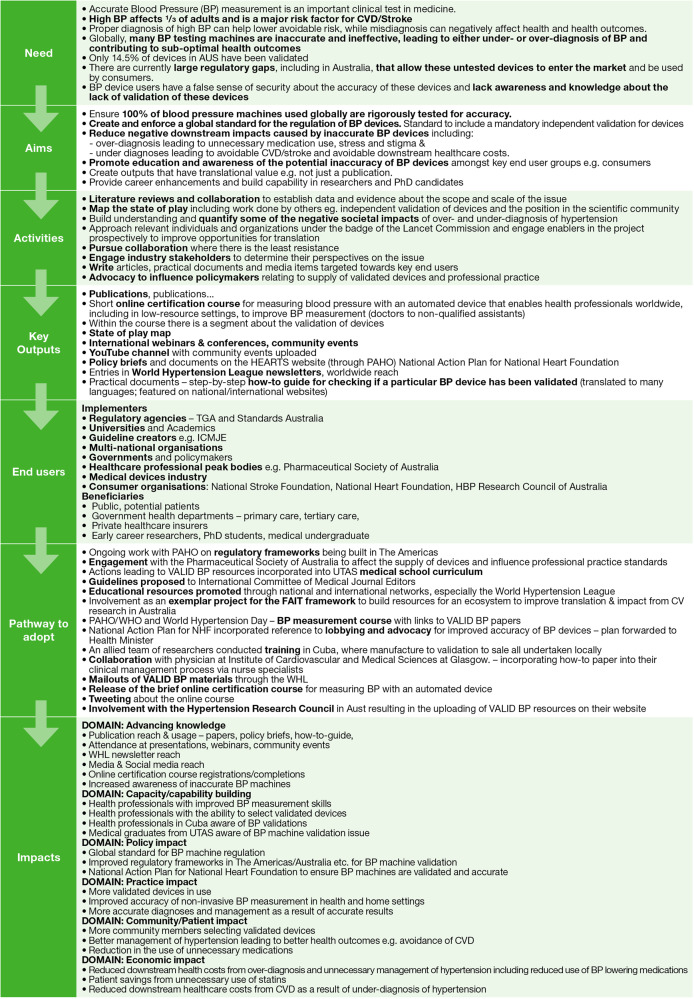
Retrospective Program Logic Model for the VALID BP Project.

To inform the costs and benefits for the CBA, a retrospective program logic model for the entire VALID BP Project was developed collaboratively by the project lead (JS) and the independent subject-matter assessors (ZD and SR). Project documentation and publications were used as references. The program logic model maps the need for the VALID BP project through to its anticipated impacts, helping to determine the costs (activities and outputs) and benefits (achieved and aspirational impacts) for the VALID BP project (Fig. 1).

### Measuring costs

The broad cost categories for the VALID BP project included research-related labour, research-related non-labour costs to cover a range of research and translational activities as previously described, and the cost of implementing a comprehensive BPMD validation programme and training health providers on the proper use of BPMDs. Research-related costs were based on historic recollection of actual time expended and other expenses relating to the VALID BP project till end 2020.

#### Research costs

##### Labour

Contributions for all the key VALID BP investigators, collaborators, and students were costed using a micro-costing methodology. Although activities were global, separating the costs by country was impossible, so the costs for all researcher time (globally) was included in the model, even though the benefits being monetised only relate to Australia. The approximate hours of researcher time (2014–2020) and the approximate wage level (converted to Australian academic wages for all positions) was used to determine the value of research labour. All researcher time for the VALID BP project was covered by the host institutions in conducting their specific tasks and not by specific grant income related to the project. Academic wages were costed using the Australian National University Academic Staff Salary Schedule[24]. Additional costs of 30% to cover superannuation contributions, payroll tax, workers compensation, and leave loading [25] and overheads of 27% to cover the in-kind contribution from the various institutions where team members were based, were added to the wage costs. These overheads covered utilities, infrastructure, and research support such as access to information technology and services provided by academic institutions. The historical research hours for the VALID BP project were allocated to systematic reviews, writing of manuscripts, development of the online BP validation course and other resources, media and communications, project meetings, training sessions and advocacy activities. Table 1 contains details of all historical labour costs.

**Table 1:** Research labour costs.

Personnel involved	Time	Hours	Academic level	Payrate	Oncosts (30%)	Oncosts and overheads (27%)		Total cost
Scientist, Australia	0.2 days/week from Dec 2014 to June 2018	750	Level E	$95	$124	$157		$117,634
	0.5 days/week since June 2018							
Ph.D. student and postdoc fellow, Australia	0.3 days/week Jan 2015–Sep 2018	624	Ph.D. stipend	$60	$78	$99		$61,813
MBBS Honours student, Australia	4 days/week Feb to Oct 2019	848	Honours student	$41	$53	$68		$57,402
Clinician/academic, Canada	0.3 days/week since June 2018	250	Level E	$95	$124	$157		$39,211
Clinician/academic, Canada	0.3 days/week since June 2018	250	Level E	$95	$124	$157		$39,211
Clinician/academic, Denmark	0.1 days/week since June 2018	83	Level E	$95	$124	$157		$13,018
ISH President, scientist, South Africa; moved to Australia	0.1 days/week since June 2018	83	Level E	$95	$124	$157		$13,018
Clinician/academic, Scotland	0.1 days/week since June 2018	83	Level E	$95	$124	$157		$13,018
Staff from an international institution	0.2 days/week since Dec 2018	166		$120	$156	$198		$32,888
Consultant from an international institution	0.4 days/week since Oct 2019	166		$95	$124	$157		$26,036
Various other individuals in various areas are involved but not significant		100		$60	$78	$99		$9906
			Total salaries					$423,156
Grants	For all incidental expenses including travel		Total non-salary					$48,681
			Total historical research costs				$471,837	

**Labour assumptions:**

1. Considering this work is ongoing, it is assumed that future research costs will be required to realise the benefits of VALID BP. Given that most of the historical research costs were expended between 2018 and 2020 (3-year period) it is assumed that the equivalent of an additional $141,052 per year in labour costs would have to be invested to maintain the momentum from this work and allow time for the realisation of 100% validation of BPMDs in Australia by 2028.
2. It is also assumed that it will take 8 more years to achieve universal validation in Australia. For each additional year, the research costs are expected to increase proportionally based on the consumer price index (CPI), but for the purposes of this preliminary model, these increases have been excluded.

##### Non-labour

Two grants totalling $48,680.62 were awarded to the VALID BP project between 2018 and 2020 and these were used to cover non-labour expenses, including travel costs (valued at $19,420), incidentals, and publishing costs.

These costs do not account for the VALID BP project work after 2020 so projections to 2028 have added an additional $48,680 per year (inflated for the purposes of remaining conservative) in non-labour costs to achieve the goal of 100% validation. This brings the project research costs up to $189,733 per year for 8 years.

#### Implementation costs

##### BPMD validation

The costs of the scientifically recommended method for validating BPMDs involve a strict research protocol which include a sample of 85 people and a cross-section of patients with different arm sizes, BPs, and gender balance [5]. The estimated cost for this method of validation was obtained from a US study published in the Journal of Clinical Hypertension [26]. The highest estimated costs were used, converted into Australian dollars and adjusted to 2022 values using the GDP deflator taken from the Australian National Accounts [27]. The total cost of a validation study was estimated at $59,340 per device model. Some manufacturers have already validated their main devices, so the burden on individual manufacturers will not be evenly spread, with some manufacturers being affected more significantly than others. Due to a lack of publicly available data on industry costs, sales, and profits of BPMDs and for the purposes of this protocol, it was assumed that the total costs to the industry for the change to full validation are included in the estimated validation cost per model. An additional 10% was added to cover any potential regulatory costs applied by the Therapeutics Goods Administration (TGA) for checking and approving device validation.

##### Regulatory change

A key translation pathway to enact effective change in the use of clinically validated BPMDs is through regulation and legislation. This would involve making BPMD validation part of the regulatory and legal requirement for registration of such devices for sale in Australia. It was unfeasible to micro-cost this activity, so existing data was used to estimate the potential cost, recognising that the journey to regulatory and legislative change is unique in every instance and unlikely to be identical in cost. A Health Research Council of New Zealand study on the cost to governments of enacting public health legislation (and deemed to be “generally applicable to other developed countries”) found that the average cost of a new regulation in New Zealand from the proposal stage through every step in the legislature (converted to Australian dollars at 2022 values) is $526 816 (95% UI: 386,602 to 1,163,500) [28] For this analysis, we have assumed that the cost in Australia is similar given the two countries have very similar constitutional and governance systems. Due to a lack of data and potential variability, we have assumed that the cost of lobbying for legislation is covered under the ongoing research costs we have projected to 2028. This legislation cost was only accounted for once in the modelled projections, in 2025, when it is feasible that advocacy efforts may have created sufficient traction for this new legislation to be enacted. This would allow a further 3 years to achieve 100% validation of BPMDs in Australia and phase out nonvalidated BPMDs already in circulation.

##### Regulatory infrastructure

The Australian TGA operates through a cost recovery framework associated with the registration and listing of medicines and medical devices. The TGA currently lists all BPMDs irrespective of whether they are validated or not. This framework generates the bulk of the agency’s funding through fees and charges paid by the manufacturers of products that are requesting approval[29]. The assumption was made that any increases in costs to the TGA or the Australian government associated with a requirement to approve only clinically validated BPMDs, would be passed on to the manufacturer and already included in the addition to validation costs for these manufacturers.

##### Costs to consumers

It is likely that BPMD manufacturers will pass on the costs of BPMD validation to Australian users, with these costs being borne by practices and consumers. However, these costs remain in the initial validation cost per model plus 10% estimate for industry and given the societal perspective, are not included again to avoid double-counting. In Australia, BPMDs (upper arm or wrist cuff devices) vary in price from $53.00 to over $630.00, with the more expensive devices generally targeted at the clinical facilities rather than personal use. Unfortunately, there is no data on the number of these devices currently on the market and potential phasing-out pathway for nonvalidated devices (compulsory versus optional and subsidised versus un-subsidised). Without this data, the costs of replacing all nonvalidated BPMDs cannot be accurately projected and were therefore omitted from the current analysis.

##### Training costs

To maximise the benefit from BPMD validation in Australia, healthcare team members need to be educated and upskilled on proper BP measurement technique. The online certified training course developed as part of the VALID BP project activities [17] takes roughly an hour to complete (including the test) and certification can be updated every six months. We have used the Australian average weekly earnings of $1713.90 per week or $55.70 per hour, including 30% additional costs and allocated two hours to complete the certification yearly. Around 565,753 health practitioners (excluding dental practitioners) were registered with the Australian Health Practitioner Regulation Agency in 2018. From 2013 to 2018, there was a 16.3% increase in the number of health practitioners, which equates to an average increase of 3.26% per year. We applied these labour costs to an estimate of the cohort requiring training in each subsequent year.

##### Scenario analysis

To estimate the downstream benefits of improved accuracy from BPMDs, we modelled and projected costs and benefits based on currently available and comparable data sources. Three different impact scenarios were tested, based on a projected trajectory for the VALID BP project from 2020 to 2028. These scenarios are all aspirational and based on estimates.

● **Scenario A**: A modest increase in the percentage of validated BPMDs caused by modest impact with policymakers, physicians, pharmacists, and consumers. Increase in the rate of validated BPMDs from 14.5% to 20% by 2021.

●**Scenario B**: Validation of all BPMDs carried by major Australian pharmacy chains due to work with the Pharmacy Guild. Validation of 60% of all BPMDs by 2025 when it is assumed that a new regulation requiring universal validation of all BPMDs on the Australian market would come into play.

●**Scenario C**: Validation of 100% of devices in Australia by 2028 including replacement of all non-validated devices currently in use.

Together, these scenarios create a form of sensitivity analysis that provides a projected minimal impact, a projected mid-point, and a maximum possible impact occurring at 100% validation, given the likelihood that there will still be nonvalidated devices in use despite the best efforts by government and industry to phase them out. It is also important to note that any changes to the accuracy of BPMDs will not rest solely with this project and that only a proportion of the benefits can be attributed to the VALID BP project.

### Modelling benefits

#### Changes to hypertension prevalence based on testing

From interviews with healthcare providers in Australia and official Australian population data [30] we derived an informed estimate of how many people over the age of 18 present for a GP consult and have a BP test every year. At baseline, this is ~18.5 million. We used a decision tree model to vary the number of people who would be under- and over-diagnosed at the different levels of device validation, and the estimated societal benefit from improving validation rates based on a reduction in unnecessary medication use and savings from avoided downstream health care costs and reduced productivity.

Currently, only 14.5% of clinically relevant upper arm and wrist cuff BPMDs are validated out of a total of 312–446 models sold in Australia. Based on the results of Akpolat et al., we also assumed that only 15% of non-validated BP devices provide sufficiently accurate measurements compared with more rigorously validated devices [10].

Assumptions underpinning the benefit analysis:

● If models are not within 4 mmHg, then they are under or overestimating BP by at least 5 mmHg [5, 6].

● In Australia, 85% of all nonvalidated models and 32% of all validated models are measuring blood pressure with an error of 5 mmHg or greater [14].

● Based on available data, the rate of overestimation (56%) is slightly higher than the rate of underestimation (44%)[31].

● All 440 models have an equal market share

● That the proportion of validated and non-validated devices currently in use is the same in both home and clinical settings

● That BPMD devices used in both home and clinical settings have an equal contribution to the overall burden of misdiagnosis

● The total number of models will remain constant with no new models entering the market between 2020 and 2028.

Supplementary file 1 details how the number of Australians being over and under-diagnosed were calculated based on the projected proportion of validated devices.

##### Medication costs due to overdiagnosis

In Australia, the annual cost of a common daily anti-hypertensive medication, Lisinopril, is listed as a subsidised medication on the Pharmaceutical Benefits Scheme[32]. The cost to consumers of the lowest dose (5 mg) assumed to be the most likely dose for someone who has normal BP (<140/90 mmHg) and is over diagnosed by up to 5 is $20.15 per pack of 30 tablets. The cost to the Government for subsidising the medication is $14.44 per pack of 30 tablets which is $175.69 per year and the cost to the patient for 365 tablets is $245.16 per year totalling $420.85 per year to both the Australian government and patients. Based on population health survey data, the assumption is that only 35% of patients diagnosed with hypertension take daily anti-hypertensive medication[33]. The assumption is also made that once commenced, patients would take these medications for life.

#### Health system savings by reducing missed diagnoses (underdiagnosis)

In 2009, it was estimated that hypertension and its consequences generated direct costs to the Australian health care system (medications, hospitalisations, emergency care etc.) of $1.8 billion over the life cycle of a given cohort, which translates to $2.27 billion in 2020 dollars (adjusted for inflation) [1]. To reduce ambiguity in the concept of a “life cycle cohort” that is found in the data, a simplified assumption was made that a cohort spanned 64 years (from 18 to 82— average life expectancy of an Australian). This means that with a highly conservative estimate, hypertension and related consequences in Australia costs the health system approximately $35,468,750 per year [34].

A Mexican study estimated that 37% of the healthcare cost of hypertension is spent in direct treatment and 63% on treatment of complications and sequalae such as myocardial infarctions and stroke [35]. When applied to our example, $22.3 million of the costs per year can be attributed to downstream costs from untreated hypertension, as a highly conservative estimate. We know from a life table monitoring study that in Australia, 61.4% of people with hypertension are left untreated [36]. Data from the AIHW confirms that 5.9 million Australians have hypertension[1]. Of these, 3.7 million (61.4%) are untreated costing the health system an average of $6.08 per person in downstream costs. This is the figure we have used to calculate the downstream healthcare costs for the cohort with undiagnosed hypertension for whom treatment is delayed.

#### Productivity costs due to hypertension

Over the working lifetime of a given Australian population cohort (ages 20–69), hypertension causes an estimated loss in economic productivity of $137.2 billion by reducing people’s capacity for work, while optimal treatment of all patients would save $76.4 billion over the working lifetime of the cohort [36]. These estimates were generated in 2009, so the savings adjusted to 2022 dollars would equal $96.15 billion over the working lifetime of the cohort. Given the working lifetime is for ~49 years from age 20–69, that equates to a saving of $1.96 billion annually. The potential lost productivity from underdiagnosis of hypertension equates to $556 million gross domestic product (GDP) or $736 per person per year. A key assumption is that of those who receive an accurate diagnosis and are identified as being hypertensive, only 35% are adequately treated and controlled. This reduces the potential savings by 65%.

## Results

The results from the modelled CBA are presented in tabular form. Table 2 presents the consolidated costs for the VALID BP project, considering the three scenarios. As evident, the research costs are negligible when compared to the implementation costs which increase exponentially as more devices are validated and more health providers are trained, peaking at between $39.4 and $48.1 million to achieve 100% validation of new devices sold, 68% accuracy of readings, and 100% competency in proper BP measurement technique by health providers.

**Table 2:** Consolidated research and implementation costs with projections till 2028.

	Baseline	Scenario A (20%)	Scenario B (60%)	Scenario C (100%)
Device validation		2021	2025	2028
Number of wearable models in Australia	446	446	446	446
% Validated (actual and aspirational)	14.3%	20.0%	60.0%	100.0%
Number of models requiring validation	0	89	268	446
Number already validated	64	64	89	268
Number of models not yet validated	382	25	179	178
Cost to validate each model	$ 59,340			
Plus TGA assumed cost of 10% of validation cost	$ 65,274			
Cost to achieve 100%, 60% and 20% validation		$ 1,644,905	$ 1,657,936	$ 1,618,772
BP certification cost of training				
Australian average weekly earning	$ 1,713.90			
Wage per hour	$ 42.85			
Wage per hour plus on cost	$ 55.70			
Wage for one hour per year	$ 111.40			
No of practitioners in 2018	$ 65,753			
No of practitioners 2021 with 20% trained	$ 124,216	$ 13,838,178		
No. practitioners in 2025 with 60% trained	$ 383,716		$ 28,909,162	
Practitioners in 2028 (100% trained)	$ 693,726			$ 34,536,201
Average cost of a new regulation from proposal stage through every step in the legislature before it is implemented (once only)			$ 526,816	
Total Implementation costs	$ 1,893,950	$ 15,483,083	$ 40,567,099	$ 6,154,973
Research labour and non-labour costs	$ 471,836	$ 661,570	$ 1,420,500	$ 1,989,699
Total research and implementation costs to achieve each scenario (*n* = 446)	$ 2,365,787	$ 16,144,653	$ 41,987,599	$ 48,144,671
Total research and implementation costs to achieve each scenario (*n* = 379)	$ 2,365,787	$ 15,269,981	$ 39,363,584	$ 43,771,313
Total research and implementation costs to achieve each scenario (*n* = 312)	$ 2,365,787	$14,395,310	$ 36,739,569	$39,397,955

Table 3 presents the detailed benefits, also considering the three scenarios and including a sensitivity analysis to account for various levels of attribution (20%, 50%, 75%). The greatest benefit is in savings from unnecessary medication, followed by productivity savings. Given the likelihood that device validation is one of many strategies and cannot guarantee accuracy of devices nor that patients accurately diagnosed will receive appropriate care, the defensible position would be to assume a 20% attribution of benefits to the VALID BP project.

**Table 3:** Consolidated costs and benefits with projections till 2028, assuming up to 100% validation.

	Scenario A	Scenario B	Scenario C
% BPMDs validated and practitioners trained	20%	60%	100%
Year	2021	2025	2028
Total research costs	$ 661,570	$ 1,420,500	$ 1,989,699
Total implementation costs	$13,733,740–$15,483,083	$ 35,319,069–$40,567,099	$37,408,257–$46,154,973
Total research & implementation costs	$ 14,395,310–$16,144,653	$ 36,739,569–$41,987,599	$ 39,397,955–$48,144,671
Medication saving in total	$ 16,875,493	$ 190,232,823	$ 541,549,886
Healthcare savings in total	$ 163,748	$ 1,845,898	$ 5,254,875
Productivity saving in total	$ 6,936,273	$ 78,190,706	$ 222,591,275
Total benefits (100% attribution)	$ 23,975,513	$ 270,269,427	$ 769,396,037

Table 4 presents the cost-benefit ratios for each of the three scenarios further segmented by the three levels of attribution. At a conservative attribution of 20% to VALID BP, the project can expect a $1.14–$1.30 return for every dollar spent with 60% validation achieved in 2025 and between $2.70 and $3.20 worth of benefit with 100% validation achieved in 2028.

**Table 4:** Cost-benefit ratios for the three scenarios, incorporating a sensitivity analysis.

	Scenario A	Scenario B	Scenario C
% BPMDs validated and practitioners trained	20%	60%	100%
Projected Year	2021	2025	2028
Cost	$ 1.00	$ 1.00	$ 1.00
Benefit (75%) Max	$ 0.98–$1.10	$ 4.27–$4.88	$ 10.12–$12.36
Benefit (50%) Average	$ 0.66–$0.74	$ 2.85–$3.25	$ 6.74–$8.24
Benefit (20%) Min	$ 0.26–$0.29	$ 1.14–$1.30	$ 2.70–$3.30

## Discussion

The VALID BP Project is a translational research project that grew organically through a mutual interest in ensuring the accuracy of BPMDs. BP measurement has an important role to play in avoiding over and under-diagnosis and mismanagement of hypertension. Using well-documented assumptions and projections of the potential costs and benefits of both device validation and better-trained users of BP devices (over an 8-year period to 2028), the analysis in this study suggests a potential return on investment in VALID BPs research, education, policy change and industry engagement activities of around 30 cents for every dollar invested after 2 years, rising to $1.14–$1.30 after 5 years and $2.70–$3.20 after 8 years if all identified hypertension is correctly managed.

This study applies a research translation and economic impact lens to a project with potential for broad societal impacts with corresponding economic benefits if implemented comprehensively. The analysis gives decision makers, in both grant and healthcare funding, information on the likely consequences of supporting this line of research and implementation. It also provides researchers with information about the potential returns for ongoing investment in translation and implementation activities. In turn this informs investment considerations to shift levers such as legislation and training programmes. Since FAIT is designed to present research impact in a way that is accessible to all stakeholders, including legislators and the general public, translating costs and benefits into a comparable ratio across research projects is valuable. This “societal” perspective for conducting CBA is particularly important for evaluating health-related research as the end products of this work can influence not only the way healthcare is delivered but downstream impacts such as productivity gains through having a healthier community and workforce.

While health and research funding decisions should not be made exclusively based on the results of an economic analysis, measures of economic impact are becoming more important in decision-making processes and therefore, it is critical that the economic methodology for assessing projects with broad societal impact be improved[37]. To this end, improving available data for economic evaluations is one of the key challenges to be overcome. Research leaders that anticipate broad societal impact from their research should consult with health economists upfront and incorporate a plan to capture the data and evidence of attribution required for appropriate economic evaluation of their research. This plan should also include consideration for a counterfactual scenario, which can strengthen the attribution of impacts to the research.

Planning a research project with the pathway to impact in mind can also help to clarify what future translation activity is required. Using the example of the VALID BP project, if analysis could reliably estimate how many Australians would need to replace their nonvalidated devices with validated models, and at what cost, policymakers would be better equipped to design evidence-based responses such as buy-back or rebate programmes. This planning process can also help to determine important avenues for concurrent research, such as determining the real-world effects of training and certification on actual blood pressure measurement practices, both in the clinical and non-clinical setting.

A key recommendation of this study is the potential expansion of the training and certification component of VALID BP to cover consumers, particularly those monitoring their own blood pressure at home as part of their ongoing management. An accessible consumer training resource would help improve overall BP measurement accuracy.

### Limitations

Although the science around valuing the returns on research investment is constantly evolving, the evaluation of the potential returns on the conduct and implementation of outcomes from health and medical research is still fraught with challenges. Various theoretical debates continue to surround even well-established methodologies such as health technology assessment, which is generally conducted for specific products using accepted principles and can often rely on sound clinical data but which is often expensive to collect [38].

Economic analysis for a project like VALID BP, which involves not only medical products but also knowledge building, advocacy, and capacity building, adds further methodological complexity. This includes questions about how to meaningfully monetise benefits like capacity and capability building, and how to accurately attribute outcomes and benefits to the research and translational activities when there are many other initiatives at various levels targeting the same outcomes and chasing the same downstream benefits.

In keeping with good practice, our cost-benefit analysis has outlined its assumptions in an explicit, up-front manner. However, the complex and exploratory nature of this work demands an additional statement on the broader limitations of this study. Primarily, the largest limitation of the modelling is the lack of available data regarding important considerations like the market share and usage patterns of the 312–446 BPMD models available for purchase in Australia. This includes the lack of data on possible differences in the level of BPMD validation between devices used for home monitoring compared with those used in clinical settings or the number of each model in use in the different settings. This lack of data required some strong assumptions, such as the assumption that the proportion of validated and non-validated devices currently in use is the same in both home and clinical settings, and that devices used in both settings have an equal contribution to the overall burden of misdiagnosis. This may not be the case, but given the lack of more granular data, there is no way of knowing. Similarly, the evidence for the impact of validation on accuracy is somewhat weak, with only one detailed study examining this phenomenon. Together, this means the magnitude of (adverse) effect on misdiagnosis cannot be exactly quantified. To account for this, we followed a detailed process for selecting assumptions for the values used in the model:

1. All our assumptions are based on the best available data we were able to source from the literature, at the time.

2. We worked closely with the international VALID BP team who are recognised experts in all aspects of BP measurement and validation and co-authors on the paper.

3. We also worked with leading cardiologists in Australia who have helped “ground-truth” the assumptions for the Australian context and are also co-authors on the paper.

4. Where variables in the model were uncertain, we managed the uncertainty using sensitivity analyses which creates a range of values with the understanding that the true value lies somewhere between the upper and lower limit.

Some of this missing data could feasibly be collected over time and then applied retroactively to this same analysis to improve accuracy, but aspects like the effectiveness of advocacy will always be difficult to estimate. The current analysis is bound by these limitations, and although the model used is only intended to offer possible projections of costs and benefits, it is critical to consider its outputs as fundamentally imperfect. Tools like the CHEERS checklist are valuable to ensure optimal methodological quality and have been applied to this study [39] but require refinement to better account for the complexities of translational health research. This could include, for example, further guidance on how to recognise and measure serendipitous research outcomes.

Until the existing issues can be resolved, economic analysis should only form one part of the research impact assessment process. For projects like VALID BP where an economic protocol with assumption-based modelling is currently possible, models like FAIT that also incorporate impact metrics and qualitative narratives of the pathway to impact can serve to bridge gaps in reliability and present decision-makers with a well-rounded understanding of the project’s societal impact while actively integrating the increasingly necessary economic component. FAIT was applied to VALID BP, using seed funding provided by the New South Wales Office of Health and Medical Research, and has been presented as an exemplar for resources created to support the application of FAIT to cardiovascular research projects. The complete FAIT application to VALID BP can be viewed at https://hmrihre.thinkific.com/ under Stream 3: Implementation Research and provides a comprehensive record of the impacts of VALID BP. A summary of the results from the other two FAIT methods appear in Supplementary file 2 [20].

## Conclusions

Overall, the cost-benefit analysis conducted in this study offers valuable insight into the potential societal impact of VALID BP project activities, especially when integrated into the broader Framework to Assess the Impact from Translational health research. The results suggest that the project’s work towards increasing the number of properly calibrated and validated blood pressure devices is an economically sound investment for improving hypertension diagnosis and management, including reducing overdiagnosis and underdiagnosis of hypertension and improving cardiovascular disease management in this population. This study also offers a methodological protocol for the economic evaluation of translational research activities that highlight the current challenges for the discipline and possible improvements to research design that can help to overcome them.

## Summary

### What is known about this topic

- Most (85%) of blood pressure measuring devices (BPMDs) available for online purchase in Australia have not been appropriately validated.
- Lack of validation contributes to potentially inaccurate BP readings, leading to both over and under diagnosis of hypertension specifically when BP is close to the threshold.

### What this study adds

- An understanding of the cost of BPMD validation and policy change in the Australian context.
- An understanding of the cost of training users of BPMDs on proper technique and interpretation of results.
- A modelled cost-benefit analysis of the impact of validation of all upper arm and wrist cuff BPMDs on the Australian market and training of health providers on proper technique based on the assumption that 100% validation and 100% trained users are achieved over an 8-year period.
- A model based on assumptions, to enable the future conduct of a more detailed cost-benefit analysis using actual data.

### Supplementary information

VALID BP Decision Tree

Payback and Narrative results for FAIT Application to VALID BP

## Data Availability

Data informing this study is not included in the manuscript but may be available through a special request to the corresponding author.

## References

[1] Australian Institute of Health and Wellfare. High blood pressure. AIHW, Editor. AIHW: Canberra; 2019.

[2] Parati G (2021). Current challenges for hypertension management: from better hypertension diagnosis to improved patients’ adherence and blood pressure control. Int J Cardiol.

[3] Gabb GenevieveM (2016). Guideline for the diagnosis and management of hypertension in adults—2016. Med J Aust.

[4] National Heart Foundation of Australia. Guideline for the diagnosis and management of hypertension in adults— 2016. Melbourne: National Heart Foundation of Australia; 2016.

[5] Stergiou GS (2018). A universal standard for the validation of blood pressure measuring devices: Association for the Advancement of Medical Instrumentation/European Society of Hypertension/International Organization for Standardization (AAMI/ESH/ISO) Collaboration Statement. J Hypertens.

[6] World Health Organization. WHO technical specifications for automated non-invasive blood pressure measuring devices with cuff. WHO medical device technical series. Geneva: World Health Organization; 2020.

[7] Picone DS (2020). Nonvalidated home blood pressure devices dominate the online marketplace in Australia. Hypertension.

[8] Picone DS (2022). Validation of blood pressure devices sold globally. JAMA.

[9] Whelton PK, Picone DS, Padwal R, Campbell NRC, Drawz P, Rakotz MK, et al. Global proliferation and clinical consequences of non-validated automated BP devices. J Hum Hypertens. 2023;37:115–9. 10.1038/s41371-022-00667-z PMC11217746 35279699

[10] Akpolat T (2009). Home sphygmomanometers: validation versus accuracy. Blood Press Monit.

[11] Hodgkinson JA (2020). Accuracy of blood-pressure monitors owned by patients with hypertension (ACCU-RATE study): a cross-sectional, observational study in central England. Br J Gen Pract.

[12] Ringrose JS (2017). An assessment of the accuracy of home blood pressure monitors when used in device owners. Am J Hypertens.

[13] Jung MH (2015). Reliability of home blood pressure monitoring: in the context of validation and accuracy. Blood Press Monit.

[14] Picone DS (2020). Nonvalidated home blood pressure devices dominate the online marketplace in Australia: major implications for cardiovascular risk management. Hypertension.

[15] Sharman JE (2020). Lancet Commission on Hypertension group position statement on the global improvement of accuracy standards for devices that measure blood pressure. J Hypertens.

[16] Olsen MH, Angell SY, Asma S, Boutouyrie P, Burger D, Chirinos JA (2016). Lancet.

[17] VALID BP Project Team. New Certification Course to Improve BP Measurement. In: League WH, editor. Hong Kong; 2019.

[18] Li J, Frick G, Herberigs K, Matsumura P, Sarkis J, Verberk WJ, et al. Industry perspectives on the global use of validated blood pressure measuring devices. J Hum Hypertens. 2023;37:130–3. 10.1038/s41371-022-00717-6 35760957

[19] Picone DS, Padwal R, Stergiou GS, Cohen JB, McManus RJ, Eckert S, et al. How to find and use validated blood pressure measuring devices. J Hum Hypertens. 2023;37:108–14. 10.1038/s41371-022-00718-5 PMC9957729 35778537

[20] Ordunez P (2022). HEARTS in the Americas: innovations for improving hypertension and cardiovascular disease risk management in primary care. Rev Panam Salud Publ.

[21] Ordunez P, Lombardi C, Picone DS, Brady TM, Campbell NRC, Moran AE, et al. HEARTS in the Americas: a global example of using clinically validated automated blood pressure devices in cardiovascular disease prevention and management in primary health care settings. J Hum Hypertens. 2023;37:126–9. 10.1038/s41371-022-00659-z PMC9957723 35273326

[22] Searles A (2016). An approach to measuring and encouraging research translation and research impact. Health Res Policy Syst.

[23] Drummond MF, Sculpher MJ, Claxton K, Stoddart GL, Torrance GW. Methods for the economic evaluation of health care programmes. 4 edition, Oxford: Oxford Medical Publications, Oxford University Press; 2015.

[24] Australian National University. Academic staff salaries Canberra: ANU; 2021; Available from: https://services.anu.edu.au/human-resources/enterprise-agreement/schedule-1-academic-staff-salary-schedule-0. Accessed 15 Dec 2021.

[25] University of New South Wales. Human Resources: On Costs Sydney: UNSW; 2021 [Available from: https://www.hr.unsw.edu.au/services/salaries/oncosts.html. Accessed 2022.

[26] Brady TM (2020). Blood Pressure Measurement Device Selection in Low‐resource Settings: Challenges, Compromises, and Routes to Progress. J Clin Hypertens.

[27] Australian Bureau of Statistics. Australian National Accounts: national income, expenditure and product, In Australia Co, editor. Quarterly estimates of key economic flows in Australia, including gross domestic product (GDP), consumption, investment, income and saving, C.o. Australia: Canberra; 2022.

[28] Wilson N (2012). Estimating the cost of new public health legislation. Bull World Health Organ.

[29] Therapeutic Goods Administration. Cost recovery implementation statement, TGA, Editor. Canberra, Australia: TGA; 2021.

[30] Australian Bureau of Statistics. Population clock Canberra: ABS; 2022 [Available from: https://www.abs.gov.au/AUSSTATS/abs@.nsf/Web+Pages/Population+Clock?opendocument&ref=HPKI. Accessed 2022.

[31] Ruzicka M (2016). How accurate are home blood pressure devices in use? A cross-sectional study. PloS One.

[32] Australian Pharmaceutical Benefit Scheme, *Lisinopril*, PBS, Editor. Canberra, Australia: Australian Department of Health; 2022.

[33] Australian Institute of Health and Welfare, Australia’s Health 2016, in Australia’s health series no. 15, AIHW., Editor. AIHW: Canberra; 2017.

[34] Australian Bureau of Statistics. Statistics about life tables for Australia, states and territories and life expectancy at birth estimates for sub-state regions, ABS, Editor. Canberra, Australia: ABS; 2021.

[35] Arredondo A, Zúñiga A (2006). Epidemiologic changes and economic burden of hypertension in Latin America: evidence from Mexico. Am J Hypertens.

[36] Hird TR (2019). Productivity burden of hypertension in Australia. Hypertension.

[37] Hahn RW, Tetlock PC (2008). Has economic analysis improved regulatory decisions. J Econ Perspect.

[38] Schlander M (2020). HTA agencies need evidence-informed deliberative processes comment on “use of evidence-informed deliberative processes by health technology assessment agencies around the globe. Int J Health Policy Manag.

[39] Husereau D (2022). Consolidated Health Economic Evaluation Reporting Standards (CHEERS) 2022 explanation and elaboration: a report of the ISPOR CHEERS II Good Practices Task Force. Value Health.

