# Supplementary figures and images for: Improving the accuracy of blood pressure measuring devices in Australia: a modelled return on investment study
Source: J Hum Hypertens. 2023 Nov 8;38(2):177–86. doi: 10.1038/s41371-023-00866-2 (PMC10844083; doi:10.1038/s41371-023-00866-2)

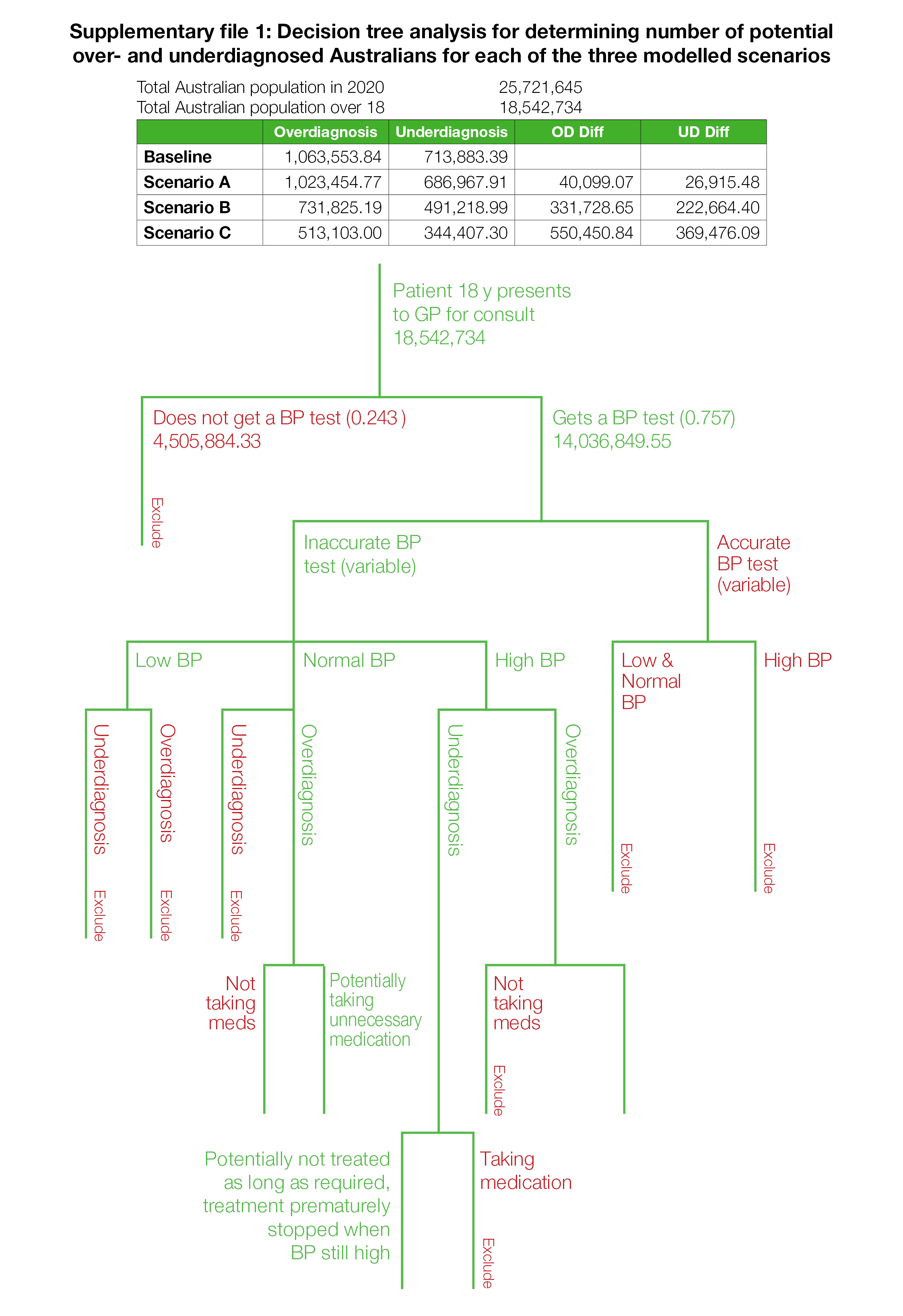

Supplement: Supplementary file 1 — VALID BP Decision Tree [file 41371_2023_866_MOESM1_ESM.jpg]
