# Supplementary material for: Improving the accuracy of blood pressure measuring devices in Australia: a modelled return on investment study
Source: J Hum Hypertens. 2023 Nov 8;38(2):177–86. doi: 10.1038/s41371-023-00866-2 (PMC10844083; doi:10.1038/s41371-023-00866-2)
Supplement: Supplementary file 2 — Payback and Narrative results for FAIT Application to VALID BP [file 41371_2023_866_MOESM2_ESM.docx]

### Supplementary file 2 – FAIT Payback Framework & Narrative for VALID BP

**PAYBACK METRICS**


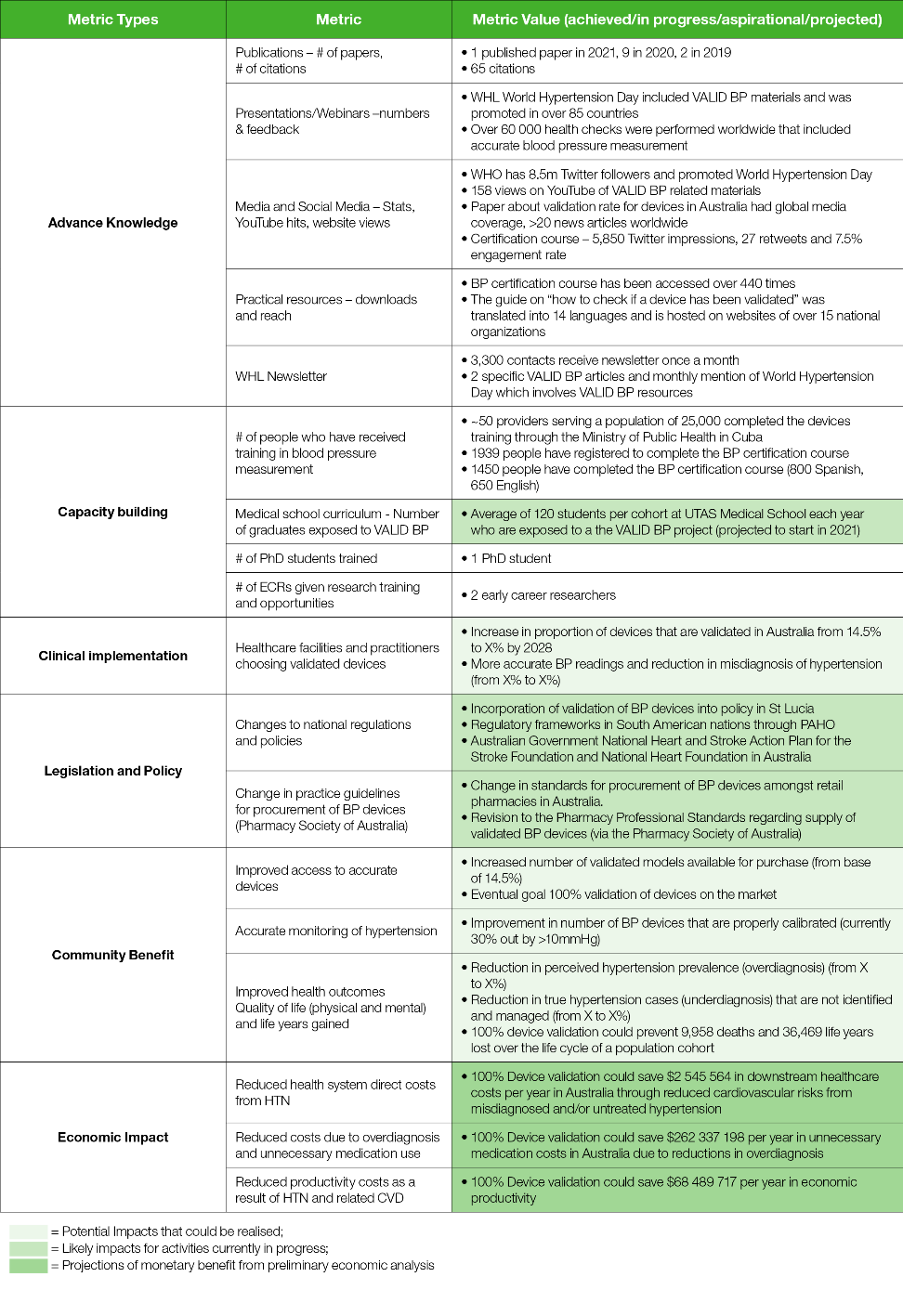


**NARRATIVE**

**Need:** Hypertension affects ⅓ of all adults globally and is a major risk factor for CVD/Stroke. Accurate diagnosis of high BP can lead to lifestyle changes or medication use that lowers avoidable risks for cardiovascular disease. Globally, many of the blood pressure testing machines in use are inaccurate, leading to either under- or over-diagnosis of hypertension. This in turn leads to avoidable demands on healthcare systems caused by unidentified and untreated hypertension, the promotion of unnecessary medication use and contribution to sub-optimal health outcomes for patients. There are currently large regulatory gaps, including in Australia, that allow untested BP devices to enter the market and be used by consumers. Device users may have a false sense of security about the accuracy of these devices and lack awareness and knowledge about the lack of validation of these devices.

**Research response:** The VALID BP Project set out to ensure that 100% of BP devices used globally are validated through creation and enforcement of a global standard for the regulation of BP devices and raising awareness and educating key stakeholders and user groups.

They conducted literature reviews and collaborated to establish data and evidence about the scope and scale of the issue and mapped the state of play and the position in the scientific community. They estimated some of the negative societal impacts of over- and under-diagnosis of hypertension, approached relevant individuals and organizations under the badge of the Lancet Commission and engaged enablers upfront to improve opportunities for translation. These enablers included the Pharmaceutical Society of Australia (to affect the supply of devices and influence professional practice standards) and the Pan American Health Organisation (to influence regulatory frameworks being built in The Americas).

They published articles in peer-reviewed journals, produced and disseminated practical guides, policy briefs and media releases and developed an online certification course for accurate blood pressure measurement, including material to ensure that validation was conducted in a rigorous and consistent manner. They used various strategies including social media to promote the course. The team presented at webinars, conferences, and community events to communicate the issue to key end users including professional associations, the public, health providers, regulatory bodies and governments, not just in Australia but globally.

The team engaged industry stakeholders to determine their perspectives on the issue and understand what part they could play in the validation process. The team used advocacy to influence policymakers to prioritise the supply of validated BP devices, both in Australia and globally, and promoted educational resources through national and international networks, especially the World Hypertension League, utilising opportunities like World Hypertension Day to disseminate information about the issue and the availability of the BP measurement course. An allied team of researchers conducted training in Cuba, where manufacture and sale are all undertaken locally. They also collaborated with physician Christian Delles from the Institute of Cardiovascular and Medical Sciences at Glasgow to incorporate a how-to paper into their clinical management process via nurse specialists. Involvement with the Blood Pressure Research Council of Australia resulted in the uploading of VALID BP resources on their website.

**Key findings/outputs:** The establishment of the size and scope of the problem, concluding that the vast majority of BP devices sold and used globally have not been properly validated through a scientific process. In Australia, only 6 per cent of BP devices on the online market are validated. Through a series of published articles, the VALID BP project highlighted the extent of this issue in many different countries, and the project also included the development of practical resources that could be used by individuals and health care providers to help them establish which devices have been validated and how to properly measure BP. The short online certification course was a key output. This material is in the process of being included in the medical curricula at the University of Tasmania. The publications and practical resources were also disseminated via digital formats and platforms and were promoted at several events co-hosted by key stakeholders, both in physical form and through online channels. There was also global dissemination of policy briefs to government and industry actors in an effort to have them implement VALID BP evidence into their policy actions and legislation. Other outputs include the State of play map; International webinars & conferences, community events, YouTube channel with community events uploaded, policy briefs and documents on the HEARTS website (through PAHO), a national action plan for the National Heart Foundation, entries in World Hypertension League newsletters, and a step-by-step how-to guide for checking if a particular BP device has been validated which has been translated to many languages; and featured on national/international websites

**Impact:** The Valid BP Project has increased awareness of the lack of validity of BP devices via publications, presentations, media and social media channels. To date there have been 1450 completions of the online BP certification course, all with improved BP management skills and the knowledge and capability to select validated BP devices. Similarly, future graduates of the medical program at UTAS and health professionals in Cuba who have undergone the training will have these same capabilities. From a policy perspective the National Action Plan for the National Heart Foundation to ensure BP machines are validated and accurate and the improved regulatory frameworks in The Americas and Australia are likely to result in improved practice in this area in the longer term. While globally it is difficult to establish the extent of the adoption of these guidelines and standards, there have been some success in smaller jurisdictions. One such example is St. Lucia where the following comment has been made by a government official “In Saint Lucia, the Saint Lucia Bureau of Standards (SLBS)used this paper as a gap analysis and going forward we will strengthen the regulatory framework for BP monitoring devices in consultation with our Ministry of Health, Ministry of Commerce, Attorney General’s Chambers, St. Lucia Medical & Dental Association and other stakeholders.”

Courses on proper measurement through VALID BP are also likely to improve accuracy and outcomes unrelated to improvements due to improved device validation, however, these impacts are more likely to be in the medium to longer term. Improved accuracy will also lead to more community members selecting validated devices; better management of hypertension leading to better health outcomes e.g. avoidance of CVD and reduction in the use of unnecessary medications including patient savings from unnecessary use of statins. There are also productivity benefits that are likely to accrue.
